# Selective shifts in mobile antibiotic resistance genes under carbamazepine exposure in wastewater microbiomes

**DOI:** 10.1093/ismeco/ycag140

**Published:** 2026-05-22

**Authors:** Eda Deniz Erdem, Bing Li, Thomas U Berendonk, Uli Klümper

**Affiliations:** Institute of Hydrobiology, Technische Universität Dresden, 01217 Dresden, Saxony, Germany; Institute of Environment and Ecology, Tsinghua University, Tsinghua Shenzhen International Graduate School, 518055 Shenzhen, Guangdong, China; Institute of Hydrobiology, Technische Universität Dresden, 01217 Dresden, Saxony, Germany; Institute of Hydrobiology, Technische Universität Dresden, 01217 Dresden, Saxony, Germany

**Keywords:** antimicrobial resistance, microbial ecology, emerging chemical pollutants, nonantibiotic pharmaceuticals, mobile ARGs

## Abstract

Carbamazepine (CBZ) is a widely used nonantibiotic pharmaceutical that frequently persists through wastewater treatment and enters aquatic environments. CBZ has been, in simplified experimental systems, reported to stimulate horizontal gene transfer (HGT), a major process of antimicrobial resistance gene (ARG) dissemination in microbial communities. Moreover, it may facilitate selection for ARGs, directly or via co-selection. However, whether CBZ significantly modulates these processes in complex microbiomes remains insufficiently characterized. To address this gap, we exposed wastewater microbial communities to a gradient of CBZ concentrations for 3 days to evaluate early responses in community composition, ARG, and mobile genetic element (MGE) dynamics. Community structure remained largely unchanged across environmentally relevant CBZ concentrations. Most ARGs showed no consistent concentration-dependent response. However, a subset of clinically relevant ARGs increased in relative abundance in a dose-dependent manner. For the beta-lactam ARGs (*bla*_CMY_, *bla*_OXA-48_, *bla*_CTX-M_), and the trimethoprim ARG *dfr*A1, this increase was significantly correlated with IncP and IncW plasmid markers and the transposable element IS26, consistent with enhanced HGT-mediated dissemination. By contrast, the macrolide resistance gene *erm*F increased independently of the tested MGE markers, suggesting direct or host-specific selection or association with a nontested MGE. The strongest shifts occurred at sub-inhibitory CBZ concentrations within the upper range of concentrations reported in wastewater-impacted environments. These findings show that CBZ exposure can indeed influence the dissemination of selected ARGs in complex microbial communities without major effects on overall community composition, highlighting the potential for nonantibiotic pharmaceuticals to shape early resistome responses to pollutants in the environment.

## Introduction

Antimicrobial resistance (AMR) poses a mounting global health threat [1]. In 2021, bacterial AMR was linked to ~4.7 million deaths worldwide, including 1.1 million directly caused by resistant infections. It is projected that by 2050, AMR could be directly responsible for 1.9 and associated with 8.2 million deaths annually [2, 3]. Most mitigation efforts against the proliferation of antibiotic-resistant bacteria (ARB) and antibiotic resistance genes (ARGs) focus on clinical and veterinary settings [4]. Despite this, there is a growing consensus regarding the necessity of understanding how the environment acts as a reservoir and conduit for AMR [4–7].

A key mechanism underlying the rapid AMR spread is horizontal gene transfer (HGT), enabling bacteria to acquire ARGs from other strains or species [8–10]. Conjugation, the transfer of plasmids among bacteria, plays a major role, as high proportions of ARGs are plasmid-borne [11, 12]. Conjugation in natural microbial communities typically occurs at low frequencies due to plasmid fitness costs and the low likelihood of encounters between donor and permissive recipient bacteria under environmentally realistic cell densities [13, 14]. A variety of biotic and abiotic factors can alter environmental conjugation frequencies in either direction [15]. Such factors include heavy metals [10], temperature [16], and various xenobiotic compounds such as microplastics [17, 18], nanoparticles [19, 20], or complex chemical mixtures like cigarette smoke [21]. Microbial interactions, such as competition or cooperation, can also positively or negatively modulate plasmid transfer frequencies [22–24]; so can exposure to antibiotics, which, in addition, serves as selective pressure for ARG-encoding plasmids [25].

Recently, nonantibiotic pharmaceuticals have been recognized as important environmental drivers of HGT. They are widespread in aquatic ecosystems due to continual discharge from households, hospitals, agriculture, and pharmaceutical production [26, 27]. Whereas microbial responses to antibiotics have been studied extensively, the impact of nonantibiotic pharmaceuticals remains an evolving field, with recent focus on effects on selection [28] and especially HGT [15, 29]. Even pharmaceuticals not classically prescribed for their antimicrobial activity can inhibit bacterial growth and enhance plasmid transfer by interacting with bacterial physiology. However, most studies to date have relied on simplified laboratory systems, often using single donor strains and high plasmid loads under conditions that diverge substantially from natural environments.

**Figure 1:**
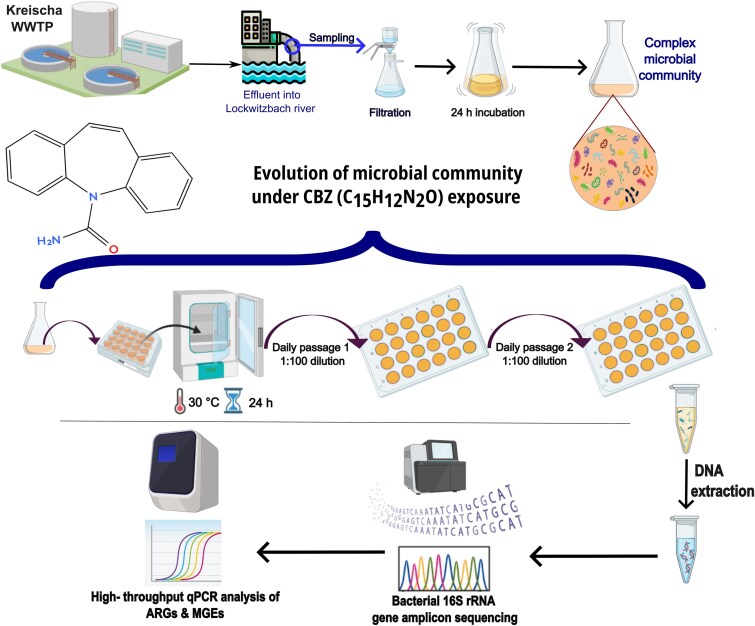
Experimental evolution framework and methods used in this study. The effluent of the WWTP was sampled to generate the model complex microbial community, which was then evolved with varying CBZ concentrations. Resulting DNA isolates were used to identify changes to the microbial community compositions and ARG and MGE relative abundances in endpoint communities.

The anticonvulsant carbamazepine (CBZ), widely used to treat epilepsy and mood disorders, represents a valuable model compound for investigating how nonantibiotic pharmaceuticals influence microbial communities and the environmental resistome. Due to its tricyclic structure (Fig. 1), CBZ persists against biodegradation and is poorly removed during conventional wastewater treatment processes reaching ng/L to low μg/L concentrations in wastewater effluents [30]. Coupled with its frequent and consistent consumption, this persistence leads to accumulation in aquatic environments, with concentrations reaching up to 3600 ng/L in surface- and groundwaters [31].

The conjugation-promoting effect of CBZ was first discovered in 2019 using single-strain mating assays with model plasmid RP4 between *Escherichia coli* and *Pseudomonas putida* [32]. The underlying mechanisms were identified as enhanced reactive oxygen species (ROS) production, increased membrane permeability, and subsequent higher expression of plasmid-borne transfer-related genes. Although further experiments extended these findings to complex microbial recipient communities, plasmid transfer was still assessed from an artificially introduced single donor strain at unnaturally high abundances, thus limiting ecological realism [33].

These findings raise an important question: Do the plasmid transfer effects of nonantibiotic pharmaceuticals observed under laboratory conditions translate to environmental contexts? Especially, the ability of CBZ to promote the spread of natural ARG-encoding plasmids has yet to be tested in environmental communities, where such plasmids are present at far lower abundances. Moreover, whether the detected plasmid transfer promotion shown for IncP-type plasmids [32, 33] is consistent for diverse types of environmental plasmids remains unknown. Finally, if beyond stimulating HGT, CBZ, similar to other nonantibiotic pharmaceuticals [28], may directly or co-select for specific ARGs, has not been explored. Such questions can best be answered using experimental evolution approaches, where microbial communities are exposed to gradients of the compound in question under controlled conditions.

Two recent studies, using fish microbiota [34] or anaerobic digestion of activated sludge [35], observed first indications of concentration- and duration-specific increases in ARG levels under CBZ exposure. Both studies focused on limited numbers of ARGs and tested limited or difficult-to-translate concentration ranges of CBZ, while also not targeting plasmids.

To overcome these limitations and assess the environmental relevance of CBZ exposure on AMR dynamics, we applied an experimental evolution framework (Fig. 1) using a complex wastewater microbial community. Communities were evolved for 3 days under a gradient of six CBZ concentrations. We then quantified changes in the absolute and relative abundance of 26 ARGs and five MGEs, including markers for three of the most common plasmid incompatibility groups found in environmental microbiomes. Thus, this study focuses on short-term, early-response dynamics rather than long-term eco-evolutionary change, providing a conservative estimate of CBZ effects. Here, we show that CBZ exposure is associated with gene- and plasmid-specific shifts in ARG and MGE relative abundance, highlighting the need to carefully consider effects of nonantibiotic pharmaceuticals in environmental AMR risk assessments.

## Materials & methods

### Wastewater effluent community

A wastewater effluent community was collected from Kreischa wastewater treatment plant (WWTP, Germany) and enriched for 24 h in Lysogeny broth (LB) after recovery from membrane filters. Detailed protocols are provided in Supplementary Methods.

### Evolution experiments

Evolution experiments were conducted in 12-well cell culture plates over 3 days with daily passages (Fig. 1). On Day 0, the 12-well plates were prepared with LB growth medium containing six concentrations of CBZ (Sigma-Aldrich, St. Louis, MO, USA) in a gradient (0, 0.05, 0.5, 5, 50, and 500 μg/ml), and the prepared wastewater community was added in 1:1000 dilution after washing with phosphate-buffered saline (PBS) and resuspension in fresh LB medium. CBZ concentrations were selected to incorporate environmentally relevant concentrations, along with those included in previous studies [31, 32]. The gradient range was expanded to 500 μg/ml to investigate the possible antibacterial activity of CBZ and the dose-dependent responses of the microbial community. Six biological replicates were conducted for each concentration and incubated at 30°C. Every 24 h, bacterial cultures were transferred to a new well containing fresh medium with the corresponding CBZ concentration in 1:100 dilution to replenish nutrients. After 72 h, samples were harvested by centrifugation, and DNA extraction was initiated immediately. Detailed extraction steps are provided in Supplementary Methods. Samples were named based on the CBZ (CBZ_0_, CBZ_0.05_, CBZ_0.5_, CBZ_5_, CBZ_50_, CBZ_500_) concentrations the communities were evolved at and are subsequently referred to using this nomenclature.

**Figure 2:**
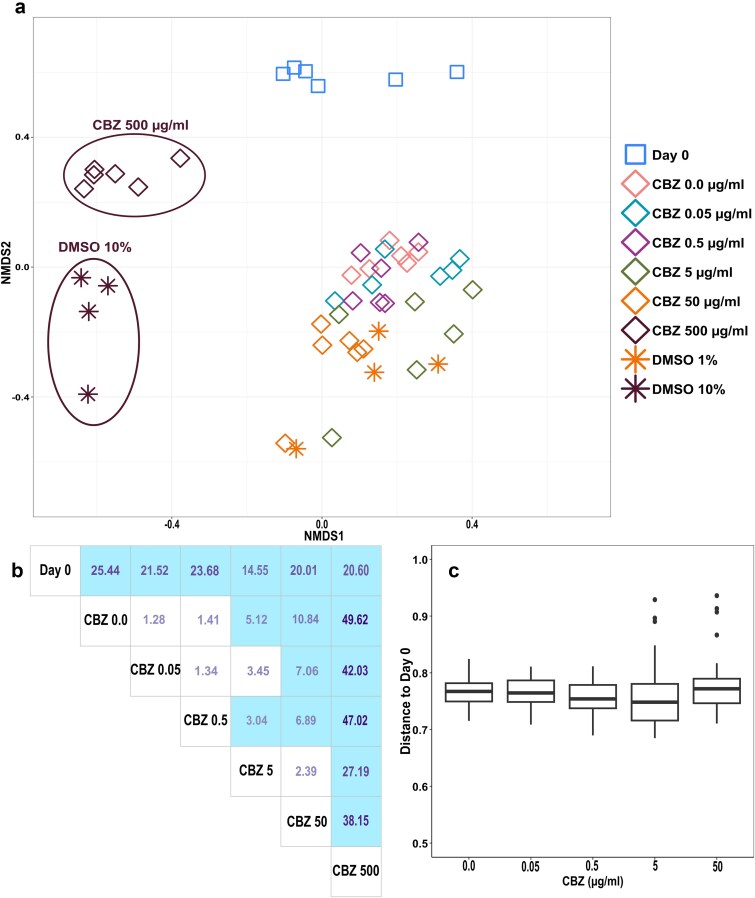
Beta-diversity analysis of the microbial communities. Community structure of evolved wastewater microbiomes based on Bray–Curtis dissimilarities of 16S rRNA gene profiles. (a) NMDS ordination of the initial community (Day 0) and all evolution endpoint samples. (b) Endpoint samples grouped by CBZ concentration and DMSO controls. Samples exposed to 500 μg/mL CBZ (CBZ_500_) and 10% DMSO (DMSO_10%_) formed a separate cluster, indicating a strong effect of high solvent concentration. In contrast, the 1% DMSO (DMSO_1%_) control clustered with treatments from 0 to 50 μg/ml CBZ (CBZ_0–50_), showing high similarity in community structure within this range. CBZ exposure, therefore, produced only minor differences relative to the large change induced by laboratory evolution itself. Statistical significance of clustering was assessed by AMOVA as indicated. (b) AMOVA post hoc pairwise comparisons of the initial microbial community and samples evolved under CBZ presence, given as *F*-statistics. Significance is indicated by a blue background (*P* < .05). (c) Bray–Curtis dissimilarity of CBZ_0–50_-evolved microbial communities to the initial community.

Because CBZ stock solutions were prepared in dimethyl sulfoxide (DMSO), exposure conditions resulted in increasing DMSO concentrations at the upper end of the CBZ gradient. The final solvent concentrations were 1% DMSO (DMSO_1%_) for the 50 μg/ml CBZ treatment and 10% DMSO (DMSO_10%_) for the 500 μg/ml CBZ treatment. To evaluate potential solvent-driven effects on community structure and resistance dynamics [36], DMSO controls were included. Significant solvent effects were observed at DMSO_10%_ compared to the CBZ_0_ communities, leading to the subsequent removal of the CBZ_500_ treatment from the analysis (*F* = 30.58, *P* < .001, Fig. 2a). Lower CBZ concentrations contained proportionally lower DMSO concentrations, resulting in solvent levels well below those reported to affect bacterial growth in similar systems [36]. These differences cannot be entirely excluded as contributing factors at low CBZ treatment levels, but the observed grouping of samples at DMSO_1%_ with those taken from CBZ_0–50_ that contained less or equal amounts of DMSO indicates that solvent effects were minimal within this range [*F* = 4.41, *P* > .05, Analysis of molecular variance (AMOVA) with Bonferroni correction for multiple testing, Fig. 2a].

### 16S rRNA gene–based quantitative PCR

To assess if overall growth patterns of the community were affected by CBZ presence during the 3-day experimental period, total bacterial abundance was estimated by 16S rRNA gene quantitative polymerase chain reaction (qPCR) using standard SYBR-Green chemistry (primer pair 338F-518R [37]). Standard plasmid [38], cycling conditions, and quality criteria are in Supplementary Methods. 16S rRNA gene copy numbers obtained were used to calculate (Eq. 1) the number of cell divisions (*X*) during the evolution experiment:


(1) \begin{eqnarray*} X={\mathit{\log}}_2\left(\frac{T_3\left(16 SrRNA\ gene\ copies\right)\times 100\times 100}{T_{initial}\left(16 SrRNA\ gene\ copies\right)}\right) \end{eqnarray*}

### Microbial community profiling

Composition and taxonomic diversity of pre-evolved and evolved microbial communities was profiled using Illumina NovaSeq V3–V4 16S rRNA gene sequencing, processed with Mothur [39] per the MiSeqSOP [40]. Operational taxonomic units (OTUs) were clustered at 97%. Full pipeline, quality filters, and subsampling details are provided in Supplementary Methods. Differences in community composition were first analysed at phylum level. Thereafter, OTU-based beta diversity was assessed via nonmetric multidimensional scaling (NMDS) based on Bray–Curtis dissimilarity. AMOVA was used to determine statistical significance of spatial clustering of the samples.

### High-throughput quantitative PCR

A subset of samples, including the CBZ-exposed microbial communities (CBZ_0–500_) and the pre-experimental, initial culture, were further analysed to quantify the potential spread of ARGs and MGEs. DNA was sent to Resistomap Oy (Helsinki, Finland) for high-throughput qPCR (HT-qPCR) analysis targeting 26 ARGs, five MGEs, and the 16S rRNA gene used for normalization using the ΔCT method [41] (Table S1). Primer sets, efficiency thresholds, and detailed processing steps are provided in Supplementary Methods. ARGs were selected to cover various antibiotic classes and include those with clinical and environmental relevance, such as those frequently found in surface waters and WWTPs, those recommended for environmental ARG surveillance [42, 43], and those that confer resistance to antibiotics classified as WHO “Watch” and “Reserve” categories according to the AWaRe classification [44]. Among the five MGEs, special emphasis was placed on the three markers corresponding to three different incompatibility groups of broad host range plasmids, IncP, IncQ, and IncW [45–47], as CBZ-based induction of plasmid transfer was previously observed for at least IncP plasmids from single donor strains [32, 33]. Because suitable and widely used marker loci with optimal coverage of the targeted group of plasmids differ between plasmid incompatibility groups, targets were selected based on established and previously validated group-specific assays (e.g. *trw*AB for IncW and *ori*T-associated regions for IncP and IncQ) [48] rather than applying a uniform gene category across all plasmid types. Further, transposable element IS*26* and class 1 integron integrase gene *intI1* were considered to be proxy targets for remaining plasmid families due to their ubiquitous nature and close association with ARG-carrying plasmids [49–51].

### Statistical analysis and data visualization

All statistical tests, data manipulations, and visualizations were executed in R Studio v2024.04 [52]. Before statistical analysis, data normality (Shapiro–Wilk) and homogeneity of variances were assessed to select appropriate tests. Correlations were evaluated using Spearman’s rank method. When needed, CBZ concentrations were log_10_-transformed, with CBZ_0_ assigned a value one log_10_ unit below CBZ_0.05_. Table S2 provides a classification of the correlation strength [53]. Pairwise comparisons were conducted using the Mann–Whitney *U* test, and multiple group comparisons with the Kruskal–Wallis rank-sum test. Unless stated otherwise, *P*-values were adjusted using the Benjamini–Hochberg false discovery rate method implemented in the *multtest* R package [54], and the threshold for significance was *P* ≤ .05. Dose–response curves and correlograms were produced with *drc* and *corrplot* [55, 56]. All remaining plots were created in *ggplot2* [57].

The rate of increase coefficient (RoI, Eq. 2) of a target at CBZ concentration *i* was calculated as described previously [58] to infer its relative abundance increase from the beginning of the evolution experiment, normalized by the shift that occurred without CBZ influence:


(2) \begin{eqnarray*} RoI=\,&{\mathit{\log}}_{10}\left(\frac{rel. abund\ in\ {CBZ}_i\ at\ {T}_3}{rel. abund\ in\ {CBZ}_i\ at\ {T}_0}\right)\nonumber\\ &-{\mathit{\log}}_{10}\left(\frac{rel. abund\ in\ {CBZ}_0\ at\ {T}_3}{rel. abund\ in\ {CBZ}_0\ at\ {T}_0}\right) \end{eqnarray*}

## Results

### Minor impact of carbamazepine exposure on microbial community composition

To examine the capacity of CBZ to facilitate AMR spread by altering HGT and selection dynamics in complex bacterial communities, an experimental evolution experiment with minimal manipulations was performed. A wastewater microbial community was exposed to six concentrations of CBZ (or their relevant solvent controls) for 3 days with daily passages in LB medium to replenish nutrients and CBZ after the communities reached carrying capacity. Although LB enrichment with daily transfers does not reproduce environmental resource conditions, this controlled design enables sensitive detection of early shifts in ARG and MGE abundances. We therefore interpret observed differences as relative treatment-specific responses rather than direct estimates of environmental effect magnitudes.

During the evolution experiment, all communities underwent significant phylogenetic changes compared to the pre-evolved community (Fig. 2a, *F* = 18.52, *P* < .0001, AMOVA with Bonferroni correction for multiple testing). Two distinct clusters of evolved communities were observed: The first cluster entailed the control communities evolved in the absence of CBZ, and all those evolved at lower and intermediate CBZ concentrations (0.05–50 μg/ml). Each treatment in this cluster was grouped significantly away from the initial samples (Fig. 2b, *F* = 14.55–25.44, all *P* < .05) and the CBZ_500_ communities (Fig. 2b, *F* = 27.19–49.62, all *P* < .05). Moreover, the DMSO_1%_ solvent control for the highest CBZ concentration in this group (CBZ_50_) grouped with the CBZ_0–50_ cluster (*F* = 4.41, *P* > .05), indicating no significant solvent effects in this concentration range.

The second cluster consisted of the communities treated with CBZ at the highest concentration (500 μg/ml; Fig. 2b, initial vs. CBZ_500_: *F* = 20.60, *P* < .05). However, these communities were subsequently removed from further analysis, as their solvent control samples DMSO_10%_ grouped far more closely with CBZ_500_ samples (*F* = 8.52, *P* = .03) compared to the non-CBZ control samples CBZ_0_ (*F* = 30.58, *P* < 0.001). Thus, only the CBZ_0–50_ cluster was analysed further.

Within this larger cluster, increasing CBZ concentrations led to a divergence of the evolved communities into two groups: Communities from the CBZ_0_, CBZ_0.05_, and CBZ_0.5_ treatment clustered together, whereas CBZ_5_ and CBZ_50_ formed the second cluster. No significant differences were detected within clusters, whereas all communities in the first cluster were significantly separated from all those in the second cluster (Fig. 2b, CBZ_0_ vs. CBZ_5_: *F* = 5.12; CBZ_0_ vs. CBZ_50_: *F* = 10.84; CBZ_0.05_ vs. CBZ_50_: *F* = 7.06; CBZ_0.5_ vs. CBZ_5_: *F* = 3.04; CBZ_0.5_ vs. CBZ_50_: F = 6.89, all *P* < .05). Nevertheless, the effect sizes of these variations in community compositions between endpoint samples were much smaller than those with the pre-experimental culture. Moreover, CBZ concentration did not correlate with Bray–Curtis dissimilarity between endpoint and initial communities (Fig. 2c, *R*_S_ = 0.06, *P* = .42, Spearman rank correlation).

### Carbamazepine did not affect community diversity but slightly reduced growth

**Figure 3:**
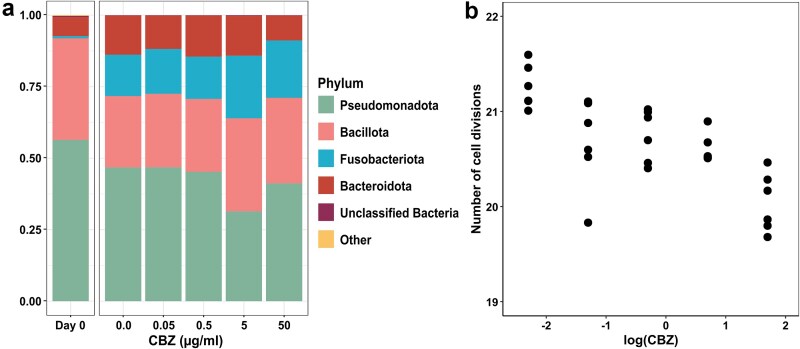
Effect of CBZ presence on the phylum-level composition and bacterial growth in the microbial communities. (a) Proportions of dominant phyla in the microbial communities. “Others” refers to phyla whose average relative abundance is <1%. (b) Number of cell divisions (Eq. 1) in the evolved communities plotted against CBZ concentration (log_10_ basis). Data points represent individual biological replicates (*n* = 6).

Eleven thousand four hundred sixty-one OTUs were identified across all samples. At the phylum level, Pseudomonadota (56.38 ± 23.21%), Bacillota (35.56 ± 17.32%), and Bacteroidota (6.72 ± 6.29%) were the most abundant phyla in the initial microbial communities (Fig. 3a). After evolution, Fusobacteriota and Bacteroidota significantly rose in prominence by 24 (*P* = .0001, Mann–Whitney *U* test) and 1.8 (*P* = .03) times compared to initial levels (Fig. 3a). Contrarily, two of the major phyla, Pseudomonadota and Bacillota, did not undergo significant changes in average relative abundance. Similarly, no significant differences among the CBZ_0–50_ endpoint samples were detectable on the phylum level (Fig. 3a). Whereas the evolution of the communities had clear effects on the beta-diversity, as well as the phylum-level distribution, only minor effects of the CBZ concentration on the diversity of the communities were noticeable.

However, CBZ exposure reduced growth during the evolution experiment. Cell divisions undergone by the CBZ_0–50_ endpoint communities showed a significant negative correlation with CBZ concentration (R_S_ = −0.78, *P* < .001, Spearman rank correlation, Fig. 3b). However, this reduction in growth was only minor, with a difference of approximately a single cell division less at the highest CBZ concentration compared to CBZ_0_. This suggests that CBZ slightly reduced growth, whereas community composition remained comparable across concentrations. Due to the minor growth effect and as community composition remained stable across evolved communities, relative rather than absolute abundances of ARGs and MGEs were subsequently utilised.

### Increasing carbamazepine concentrations are linked to higher total relative plasmid abundance

Based on these minor effects of CBZ on community composition and growth, whether it altered the relative abundance of 26 ARGs and five MGEs through either selection or modulated HGT rates was tested across the initial communities and communities evolved under CBZ exposure (CBZ_0–50_), with CBZ_500_ being excluded due to the solvent effects detected above.

**Figure 4:**
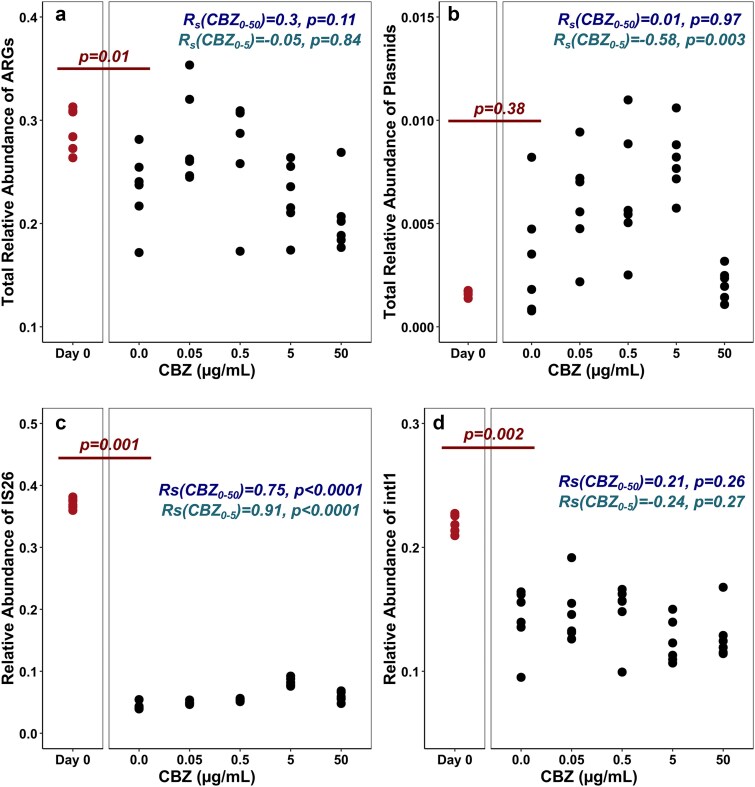
Relative abundances of the genes of interest by type. Relative abundances per 16S rRNA gene in the evolved microbial communities as a function of increasing CBZ concentrations of (a) total ARGs displayed as the sum of the relative abundance of the tested 26 ARGs, (b) sum of IncP, IncW, and IncQ plasmid markers, and (c) transposable element IS*26*, (d) class1 integron integrase gene *intI1*. Data points represent individual biological replicates (*n* = 6).

The normalised relative abundance of total tested ARGs decreased significantly by 52.27% during the evolution experiment in the absence of CBZ (Fig. 4a, initial vs. CBZ_0_: *P* = .01, Mann–Whitney *U*). Contrarily, the total relative plasmid marker abundance (0.002 ± 0.0001) was not significantly impacted by the evolution experiment in the absence of CBZ (Fig. 4b, *P* = .38). For the remaining MGEs, transposable element IS*26* and class 1 integron integrase gene *intI1,* a sharp reduction of 88.43% and 35.43% was observed in the absence of CBZ (Fig. 4c and d, both *P* < .01).

Among the evolved communities, no significant changes in relative abundance of total ARGs, total plasmids, or other MGEs were observed across any individual CBZ concentration compared to the nonexposed control (CBZ_0_ vs. CBZ_0.05–50_: all *P* > .05, Mann–Whitney *U*, False Discovery Rate (FDR)-adjusted). Moreover, no dose–response correlation of CBZ was detected when considering all CBZ concentrations except for IS*26* (total ARGs: R_S_ = −0.30, *P* = .11; total plasmids: *R*_S_ = 0.01, *P* = .97; *intI1*: *R*_S_ = 0.21, *P* = .26; IS*26*: *R*_S_ = 0.75, *P* < .0001; Spearman rank correlation). However, at the CBZ concentration range between 0 and 5 μg/ml, a strong and significant dose-dependent relationship was observed between CBZ exposure and total relative abundance of plasmid markers exclusively (*R*_S_ = 0.58, *P* = .003). Despite plasmids as the main vehicles of ARG spread correlating with CBZ in this concentration range, no correlation with total ARGs (*R*_S_ = −0.05, *P* = .84) and no correlation between total relative plasmid and ARG abundance in the dataset was detected (*R*_S_ = 0.25, *P* = .19). This pattern may reflect that only a subset of the tested ARGs is plasmid- or MGE-associated, or that CBZ influenced only specific MGEs within the community. Consequently, ARG, plasmid, and IS*26* dynamics across the CBZ gradient were investigated next on a gene-by-gene basis. As class 1 integrons (*int*I1) were not affected by CBZ, these were excluded from subsequent analysis. Their stability suggests no detectable short-term response to the tested conditions.

### Gene-dependent dose–response effects of carbamazepine exposure

To determine whether CBZ-induced spread of the individual ARGs occurred and if it was connected to the induction of plasmid or IS transfer, their fluctuations in relative abundances were evaluated. The number of detected ARGs was comparable in all evolved microbial communities (24.31 ± 0.70 of 26 tested ARGs, *P* = .57, Kruskal–Wallis rank sum), with only one ARG (*bla*_OXA-58_) discarded from in-depth analysis due to being under the detection limit for the majority of samples (detection in 28% of samples).

**Figure 5:**
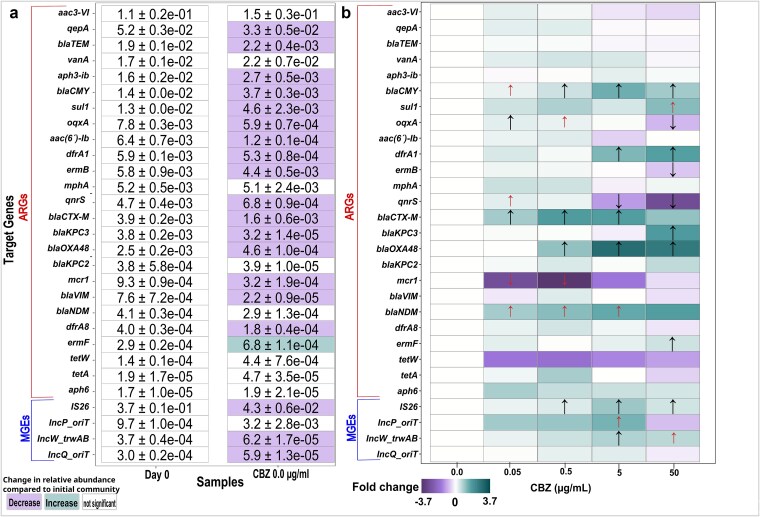
Impact of CBZ exposure on relative abundances of individual genes of interest. (a) Changes in relative abundances of ARGs and MGEs during evolution without CBZ presence. Values represent the average relative abundance of the target gene ± standard deviation between biological replicates (*n* = 6). Purple background on CBZ_0_ samples refers to a significant decrease from the relative abundance of the gene in the initial community (Day 0), whereas a significant increase is indicated by a blue background (FDR-adjusted *P* < .05, Mann–Whitney U test). (b) Heatmap of relative abundances of ARGs and MGEs of interest in the evolved communities, based on the log2 fold change of gene abundances in each replicate relative to the average of CBZ0 samples. Arrows indicate a significant shift (upward: increase; downward: decrease) in relative abundances of the genes compared to that in the CBZ_0_ communities (FDR-adjusted *P* < .05 for black arrows, 0.05 < *P* < .10 for red arrows, Mann–Whitney *U* test). Target genes are ordered by their relative abundances in the initial microbial community, from highest to lowest within their category (ARG or MGE).

Evolution alone, independent of CBZ pressure, had a measurable impact on ARG and MGE relative abundance. 16/25 ARGs, 2/3 plasmid markers, and IS*26* all decreased significantly in abundance after evolution (FDR-adjusted *P* = .003–.001, Mann–Whitney *U*, purple background in Fig. 5a). Only one ARG, *erm*F, which confers resistance against macrolides, had a 2.3-fold significantly elevated abundance after evolution (blue background in Fig. 5a, FDR-adjusted *P* = .003).

In the evolved communities, CBZ had varying effects on ARG and MGE relative abundances (Fig. 5b). For 13/25 ARGs, including the five most abundant ARGs: *aac3*-VI, *qep*A, *bla*_TEM_, *vanA,* and *aph*3-ib, no significant difference compared to the control was detected at any concentration (all FDR-adjusted *P* > .1). Only 5/25 ARGs exhibited significant relative abundance surges across the majority of CBZ-exposed communities (log_2_-fold change: 0.21–2.84, FDR-adjusted *P* = .02–.06, black and red upward arrows in Fig. 5b). Among these were four β-lactam ARGs (*bla*_CTX-M_, *bla*_CMY_, *bla*_OXA-48_, *bla*_NDM_) and *dfr*A1 that confers resistance to trimethoprim. Additionally, significant increases at only the highest CBZ concentration were detected for three ARGs (*bla*_KPC3_, *erm*F: black upward arrows at CBZ_50_, *sul*1: red upward arrow at CBZ_50_). Among the remaining four ARGs, significant decreases in abundance were detected only once at lower (*mcr*1: red downward arrows at CBZ_0.05–0.5_) or once at the highest CBZ concentration (*erm*B: black downward arrow at CBZ_50_) compared to the control. Initial increases at low and sharp decreases at high CBZ concentrations were detected twice (*opx*A, *qnr*S, Fig. 5b).

For MGEs, significant increases in relative abundance across the majority of CBZ-exposed communities were observed for IncW plasmids and IS*26* (upward arrows at CBZ_0_._5–50_, log2-fold change: 0.33–0.94, FDR-adjusted *P* = .02–.06). For IncP plasmids, an increase at intermediate CBZ concentrations (upward arrow at CBZ_5_, FDR-adjusted *P* = .07) followed by a 1.6-fold, but nonsignificant decrease in relative abundance at CBZ_50_ was detected, mirroring the previous trend for total relative plasmid abundance. Unlike for IncW and IncP plasmids, no effects on IncQ plasmids at any concentration were observed (FDR-adjusted *P* = .33–.90).

**Figure 6:**
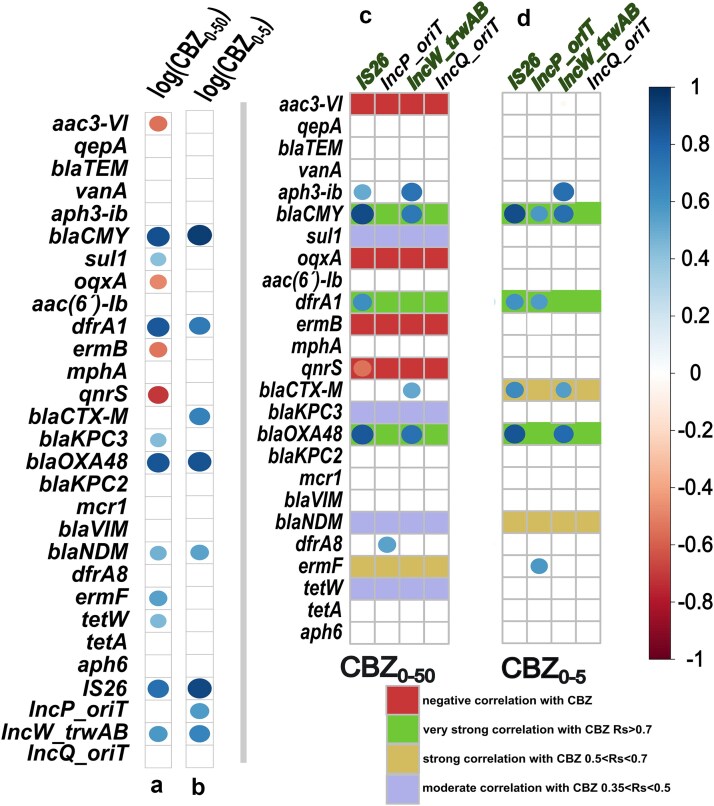
Correlations of CBZ exposure with the relative abundances of individual genes of interest, and ARG–MGE associations. Spearman correlation analyses between the relative abundances of target genes, ARGs, and MGEs and the log10-transformed CBZ concentration across the full (A, C; 0–50 μg/ml) and intermediate (B, D; 0–5 μg/ml) ranges. A and B show correlations between CBZ concentration and relative abundances of target genes, while C and D present ARG–MGE correlations, visualized as correlograms. Only significant correlations (FDR-adjusted *P* < .05) are displayed, with ARG–MGE relationships visualized as correlograms. Target genes are ordered by their relative abundance in the initial microbial community, from highest to lowest. Strong correlations between CBZ concentration and relative abundances of MGEs (as described in Fig. 6a and b and categorized in Table S2 [53]) are indicated as green legend text. The strength of correlations between ARG relative abundances and CBZ concentration (as described in Fig. 6a and b) is represented by background colour coding: green (very strong correlations, RS > 0.7), yellow (strong correlations, 0.5 < RS < 0.7), purple (moderate correlations, 0.35 < RS < 0.5), and red (negative correlations), as categorized in [53].

In the absence of a comprehensive trend for the spread of ARGs and MGEs in evolved microbial communities, Spearman's rank correlation analysis helped clarify the role of CBZ concentration-dependent dose–responses. When taking all CBZ concentrations from 0 to 50 μg/ml into account, significant positive correlations with the relative abundances of 8 of the 25 ARGs were identified (*bla*_CMY_, *sul*1, *dfr*A1, *bla*_KPC3_, *bla*_OXA-48_, *bla*_NDM_, *erm*F, *tet*W, Fig. 6a). Except for *bla*_CTX-M_, these included all ARGs for which previous increases in relative abundance were detected at several concentrations, all ARGs for which significant increases were only detected at the highest CBZ concentration and *tet*W, for which no individual sample displayed any significant change in relative abundance (Fig. 6a). The strongest positive dose–response relationships were found for *bla*_CMY_, *bla*_OXA-48_, and *dfr*A1 (R_S_ = 0.84–0.87, FDR-adjusted *P* < .05), whereas the remaining positive correlations with CBZ concentration for *erm*F, *sul*1, *bla*_KPC3_, *bla*_NDM_, and *tet*W were moderate (*R*_S_ = 0.42–0.47, FDR-adjusted *P* < .05). Negative correlations were identified between CBZ and the relative abundances of four ARGs: *aac3*-VI, *oqx*A, *erm*B, and *qnr*S (*R*_S_ = 0.49–0.71, 0.0001 < FDR-adjusted *P* < .02). Among the MGEs, very strong (*R*_S_ > 0.70) and moderate (0.30 < *R*_S_ < 0.50) positive correlations with CBZ were detected for IS*26* and IncW plasmids, respectively.

A second Spearman rank correlation analysis was performed within the range of CBZ concentrations that had previously yielded a significant relationship with total relative plasmid abundance (*bla*_CMY_, *dfr*A1, *bla*_CTX-M_, *bla*_OXA-48_, *bla*_NDM_, CBZ_0–5_, Fig. 6b). This removed the majority of moderate correlations, except for *bla*_NDM_ (*R*_S_ = 0.53, FDR-adjusted *P* = .03), including all negative correlations of ARGs. All previous strong correlations remained significant (*bla*_CMY_, *bla*_OXA-48_, and *dfr*A1, IS*26*, and IncW plasmids, *R*_S_ = 0.67–0.95, FDR-adjusted *P* < .005), whereas two new strong correlations between CBZ and *bla*_CTX-M_, as well as IncP plasmids, were apparent (*R*_S_ = 0.68 & 0.57, FDR-adjusted *P* < .05). As concentration-dependent increases for a number of ARGs and MGEs were observed, next, their dependency on each other was tested.

### Correlations with mobile genetic elements explain carbamazepine dose–response effects on antimicrobial resistance genes

To investigate whether the observed CBZ dose–response increases in certain ARGs are based on HGT, correlations between MGE and ARG relative abundance within the evolved microbial communities using the full (CBZ_0–50_, Fig. 6c) and the intermediate (CBZ_0–5_, Fig. 6d) concentration range were tested.

In the intermediate CBZ concentration dataset, four out of five ARGs with strong and very strong positive correlations to CBZ concentrations (R_S_ > 0.68, green and first yellow backgrounds in Fig. 6d) correlated with at least two of the three MGEs (IncP plasmids, IncW plasmids, IS*26*) that also displayed positive correlation with CBZ. *bla*_CMY_ positively correlated with all three MGEs (*R*_S_ = 0.58–0.89, FDR-adjusted *P* = .00001–.04), *dfr*A1 correlated with IncP plasmids and IS*26* (*R*_S_ = 0.56 & 0.61, FDR-adjusted *P* < .05) and *bla*_CTX-M_ and *bla*_OXA-48_ correlated with IncW plasmids and IS*26* (*R*_S_ = 0.55–0.86, FDR-adjusted *P* = .0001–.05). Contrarily, *bla*_NDM_ that displayed a less strong correlation with CBZ (second yellow background in Fig. 6d) did not correlate with any of the MGEs. Among the 20 ARGs that did not correlate with CBZ, in general, no positive correlations with any MGEs were detected. Exceptions were *aph*3-ib, which encodes an aminoglycoside-modifying enzyme and correlated with IncW plasmids and IS*26* (*R*_S_ = 0.75 & 0.55, respectively, FDR-adjusted *P* < .05), and *erm*F, which displayed a relatively weak correlation with IncP plasmid abundance (*R*_S_ = 0.57, *P* = .04). IncQ plasmids that were not affected by CBZ did not display a single ARG correlation throughout.

**Figure 7:**
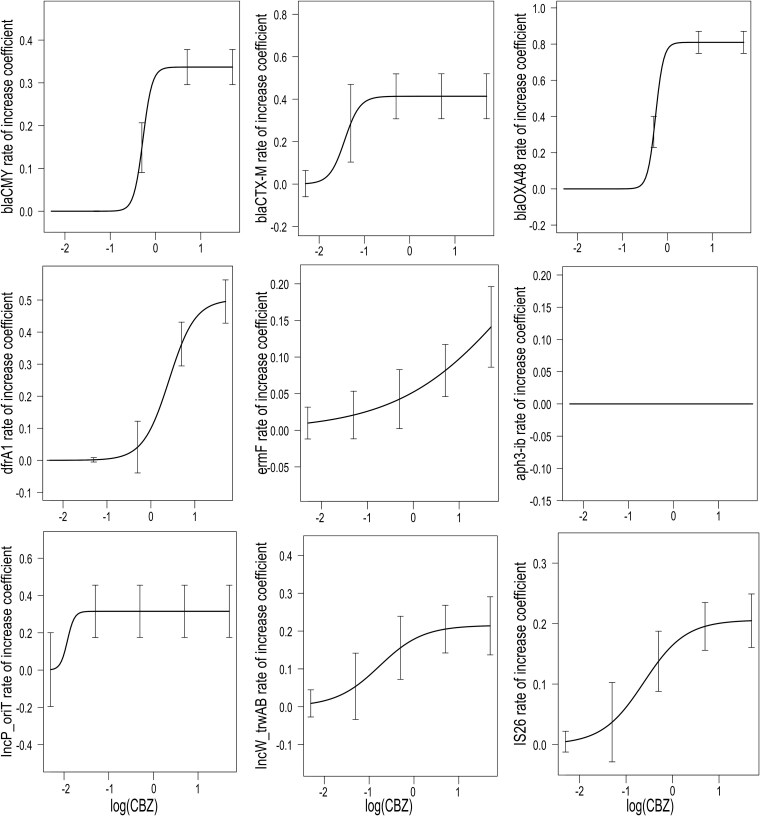
Dose–response dynamics between rate of increase and CBZ concentration for selected genes. Dose–response models built to examine the rate of increase of relative abundances (Eq. 2) of *bla*_CMY_, *bla*_OXA-48_, *bla*_CTX-M_, *dfr*A1, *erm*F, *aph*3-ib, IncP, IncW, and IS*26* markers in relation to the log10 basis of CBZ concentration. The three-parameter log-logistic (LL.3) function from the drc R package [55] was used to build the models, and the error bars represent model-based standard errors.

Similar trends were visible when investigating the full dataset, with those ARGs displaying very strong positive correlations with CBZ (green background) generally displaying MGE correlations, whereas those with only strong positive (yellow), moderate positive (purple), no (colourless), or negative correlations with CBZ mainly displaying no MGE correlations (Fig. 6c). However, in the full dataset, again, a few correlations between MGEs and ARGs that had no CBZ correlations (*aph*3-ib, *van*A, *dfr*A8, & *tet*A) were detected. These correlations may reflect plateau behavior at lower concentrations or variability at higher concentrations, so they should be interpreted cautiously. Consequently, their rate of increase during the evolution experiment (Eq. 2) was modelled as a function of CBZ dose–response (Fig. 7).

Indeed, for IncP plasmids, a plateau of the rate of increase was reached at a very low dosage of CBZ (between 0.05 and 0.5 μg/ml). Contrarily, no plateau for the rate of increase was apparent for IS*26* and IncW plasmids, although the model implicates that the maximum response may be observed around CBZ_50_. For the four ARGs that displayed strong CBZ dependence and strong MGE correlations throughout (*bla*_CMY_, *bla*_OXA-48_, *bla*_CTX-M_, & *dfrA*1), clear dose–response relationships were modelled. For *bla*_CTX-M_, a similarly low plateau concentration of CBZ to that of IncP plasmids (between 0.05 and 0.5 μg/ml) was observed. For the *bla*_CMY_ and *bla*_OXA-48_ genes, the plateau concentration was in the intermediate range between 0.5 and 5 mg/ml, whereas for *dfr*A1, similar to IS*26* and IncW plasmids, no plateau for the rate of increase was apparent with the model implicating that the maximum response may be observed around the maximum tested CBZ concentration.

For those ARGs with potentially random correlations with MGEs but not with CBZ (Fig. 6d), dose–response modelling was either impossible (*aph*3-ib) or relative abundances were increasing throughout with a steep slope, even as a response to the highest CBZ concentration tested (*erm*F) (Fig. 7).

## Discussion

Previous studies have shown that CBZ can promote plasmid-mediated ARG horizontal gene transfer (HGT), with up to 12- and 7.1-fold increases in conjugation frequency [31, 32], and may drive concentration- and duration-dependent ARG increases in complex communities [33, 34]. Our experimental evolution study builds on this foundation by testing these mechanisms under more realistic conditions. Here, despite a minor, dose-dependent growth-inhibiting effect, CBZ had no major effect on community structure or total relative ARG abundance. Rather, it selectively increased the relative abundance of specific ARGs and MGEs at subinhibitory and potentially environmentally relevant concentrations (0–5 μg/ml). Four clinically relevant ARGs displayed clear dose-dependent increases correlating with the proliferation of IncP and IncW plasmids and the transposable element IS*26*, consistent with enhanced HGT, in line with prior mechanistic studies [31–34]. However, other tested MGEs and ARGs that were not associated with any MGE markers were not significantly affected by CBZ exposure. Together, our results suggest that CBZ can influence AMR propagation in complex microbial communities through both HGT- and gene-specific selection, albeit in a more nuanced and limited manner than previous laboratory studies might imply.

Significant correlations were observed between CBZ and relative abundances of transposable element IS*26*, and conjugative IncP and IncW plasmids. Whereas previous studies reported strong CBZ-induced effects on IncP plasmids such as RP4 [32, 33], we observed that effects on IS*26* abundance were even greater. This is likely due to IS*26*’s frequent association with conjugative plasmids [51], which benefit from enhanced transfer under CBZ exposure [29, 32, 33]. Moreover, once present in a host, IS*26* can amplify within cells through replicative transposition, especially under stress conditions, independent of HGT [49, 51]. This dual capacity to spread both between and within cells may explain the particularly strong IS*26* response to CBZ.

These observations are consistent with the proposed mechanism of CBZ-induced ROS overproduction, which activates the bacterial SOS response, thereby promoting expression of conjugation-related genes such as *tra* via derepression of *kor*AB regulators in IncP and potentially IncW plasmids [29, 59–64]. Additionally, IS*26* activity may also be directly enhanced by the same ROS-triggered SOS stress cascade, as transposase expression and replicative transposition are known to respond to such stress [49]. The strongest correlations between CBZ exposure and MGE and MGE-associated ARG relative abundance were observed within the 0–5 μg/ml range. This concentration range (0–5 μg/ml) reflects the upper levels of CBZ detected in aquatic environments, including effluents, surface water, and occasionally groundwater [27, 30, 31]. Associations weakened across the full 0–50 μg/ml gradient used in this study, suggesting a potential plateau or even inhibitory effect at higher CBZ concentrations. Similar nonlinear dynamics have been reported in earlier studies [29, 32, 65]. ROS production and conjugation or transformation efficiencies increased at low CBZ concentrations but failed to rise further or even declined at higher doses. Moreover, in the case of transformation efficiencies, subinhibitory concentrations of non-antibiotic pharmaceuticals most effectively enhanced HGT, whereas excessive doses yielded diminished effects [65]. ROS production was not directly quantified in this study, due to its interpretation in complex microbial communities remaining challenging, as both baseline levels and dose-dependent responses are strain-specific [66] and not readily resolved by bulk measurements. Still, our findings support a model in which moderate ROS stress induced by CBZ promotes plasmid transfer and MGE activation, but excessive stress may suppress these processes via cellular damage, defensive regulatory systems, or metabolic burden.

However, these promoting effects appear to be MGE-specific: although IncP, IncW, and IS*26* responded clearly, mobilizable IncQ plasmids, despite their broad host range, showed no significant dose-dependent change. This is unlikely to result from a lack of compatible conjugative plasmids, needed to support IncQ transfer [67], as, for example, IncP and IncW plasmids were abundant, and, due to the natural origin of the community, likely the same is true for other conjugative plasmid groups. Instead, it suggests that CBZ-induced ROS preferentially stimulates self-transmissible conjugative systems, whereas mobilization-dependent elements such as IncQ [68, 69] are less affected in complex communities.

Similarly, class 1 integron integrase gene *intI1* did not increase in response to CBZ, despite being under *lex*A repression and part of the SOS regulon. This lack of response may reflect a regulatory activation threshold for *intI1* that was not met under CBZ-induced ROS stress. Although moderate ROS levels may have been sufficient to activate *tra* operons and IS*26* transposition, previous studies show that *intI1* requires strong, sustained SOS induction, typically triggered by DNA-damaging antibiotics like fluoroquinolones [70, 71]. Moreover, *intI1* is not self-transmissible, relying on its association with conjugative elements for HGT, and in natural communities, a substantial proportion of class 1 integrons are chromosomally encoded [72]. This chromosomal integration may decouple integron dynamics from plasmid-level conjugation effects, masking any impact of CBZ on *intI1* abundance.

Overall, these findings still emphasize that the impact of CBZ on ARG propagation is gene- and MGE-specific, rather than broad or uniform. Whereas ROS-mediated stimulation of conjugation and transposition, as demonstrated previously [31–34], can explain much of the observed HGT activity, selective pressures should not be ruled out. Notably, *erm*F exhibited a clear dose–response increase, yet did so independently of the tested MGEs, suggesting that either direct selection by CBZ, its host’s adaptation to lab conditions, or association with a nontested MGE may have driven its proliferation. Similar selection dynamics for resistance determinants were observed for other nonantibiotic compounds [28]. Distinguishing between CBZ’s effects on conjugation versus selection remains a key future challenge.

This study builds on previous work by extending the investigation of CBZ-driven AMR dynamics from simplified laboratory setups to a complex, natural microbial community, while applying environmentally relevant concentrations of CBZ across a broad gradient. Whereas earlier studies mainly focused on single-strain conjugation assays [32], relied on artificially introduced plasmids and donors [33], or focused on limited targets and concentrations [34, 35], our experimental evolution approach provides a more ecologically realistic model to assess ARG and MGE behaviour in response to CBZ exposure. There are discrepancies in the protocols of these previous studies and our current research, including variations in trial duration and the origin of the model bacterial strains; still, the temperature, media, and CBZ concentrations were consistent. Although a direct comparison is implausible, our study focused on investigating how these findings may be reflected in conditions more environmentally relevant. By quantifying both plasmid and transposon dynamics, our study advances understanding of how nonantibiotic pharmaceuticals influence HGT of ARGs in natural microbial communities.

Nonetheless, several limitations should be acknowledged. First, the observed effects of this experiment, conducted over 3 days represent only short-term responses and do not fully capture the complexity or resource constraints of natural systems. Consequently, the observed effects likely reflect initial shifts in dissemination dynamics as a direct response to CBZ exposure rather than stable evolutionary change, and longer experimental timescales or *in situ* community conditions may yield different adaptation outcomes. Higher CBZ concentrations than typically measured in surface waters were included to characterise the dose–response window; therefore, effects at the upper end of this range should not be directly extrapolated to the environment. Still, even at lower, environmentally relevant concentrations, effects were, while less pronounced, detectable. Additional subtle impacts at environmentally relevant concentrations may, however, only emerge over extended periods. Indeed, a recent aquatic mesocosm study showed that community-level responses can take weeks to months to manifest at μg/L CBZ levels [73]. Longer-term exposures under environmentally representative conditions are needed to resolve potential cumulative or latent effects on ARG and MGE dynamics. As such, the limited responses observed at lower concentrations here likely represent a conservative baseline.

Second, we inferred resistance dissemination from changes in relative ARG and MGE abundances and their correlations, without resolving specific gene–host linkages. This prevents differentiation between mechanisms such as HGT, clonal expansion, or intracellular amplification. Future studies incorporating long-read metagenomics, Hi-C sequencing [74], epicPCR [75], or multiplex-ddPCR linkage analysis [76] will enable host- and MGE-resolved ARG trajectories.

Third, the laboratory conditions under which this study was conducted, namely, nutrient-rich media and elevated temperature, may have produced a microbiome that is not fully representative of environmental communities, as some species are not culturable under these conditions. These parameters could also influence ARG and MGE mobility, since both increased nutrient availability [77, 78] and higher temperatures [79, 80] have been shown to enhance conjugation efficiency compared with the more variable or suboptimal conditions typically found in WWTPs. However, conjugation is not restricted to optimal conditions and has been shown to occur readily even under nutrient scarcity or lower temperatures [78, 79]. Although the experimental setup may have been biased towards higher transfer rates, the observed effects likely still reflect processes that can occur under environmentally relevant conditions.

In addition, the potential influence of other biotic components, such as protists or bacteriophages, was not resolved in this study. While all treatments originated from the same initial community, these components may also respond to CBZ exposure and thereby influence ARG dynamics in complex microbial systems [81–84].

Finally, although solvent controls were included at 1% and 10% DMSO, lower CBZ treatments contained proportionally lower DMSO concentrations. Minor solvent effects, therefore, cannot be fully excluded at the lowest CBZ levels, although no consistent clustering was observed at 1% DMSO in community structure analyses.

Despite these limitations, our findings raise broader questions about the evolutionary pressures that nonantibiotic pharmaceuticals may impose on natural microbial communities. For example, if long-term CBZ exposure could drive co-selection and co-evolution between ARGs and MGEs such as IncP, IncW, or IS*26*, by selectively enriching those ARG vehicles best suited to thrive under chemical stress, remains to be investigated. If so, this could shape the structure of the environmental resistome in ways not visible in short-term assays. Our results also suggest that, in addition to conjugative plasmids, MGEs like IS*26*, due to their strong and mechanistically grounded response to CBZ exposure, may serve as biomarkers of pharmaceutical-driven HGT and be useful for AMR surveillance in wastewater-impacted ecosystems.

In conclusion, our study demonstrates that the nonantibiotic pharmaceutical CBZ can selectively promote the spread of ARGs in complex microbiomes. Whereas overall community composition remained largely unaffected, CBZ exposure drove dose-dependent increases in specific clinically relevant ARGs. These increases were linked to IncP/IncW plasmids and the transposon IS*26*, consistent with the suggested enhanced HGT. In contrast, the macrolide ARG *erm*F rose independently of the tested MGEs, indicating a role for direct ARG selection or association with a nontested MGE.

These findings highlight that CBZ, and potentially other nonantibiotic pharmaceuticals [29, 32, 33, 35], can shape the environmental resistome in a targeted rather than community-wide manner, with concentrations in the range of those detected in surface waters exerting the strongest effects. Given the widespread occurrence of CBZ and other nonantibiotic pharmaceuticals in aquatic environments [27] and here and elsewhere [29, 32, 33, 35] demonstrated capacity to modulate ARG dynamics, we thus recommend their inclusion in AMR risk assessment frameworks and environmental monitoring programs [5, 85, 86]. However, unlike for antibiotics, where defining predicted no-effect concentrations for selection (PNEC_res_) is straightforward [87], defining such PNEC_res_s for compounds that mainly affect AMR through HGT modulation or co-selection remains more challenging. Still, surveillance systems should be expanded to consider not only antibiotic usage but also the broader pharmaceutical context that shapes resistance dissemination in real-world microbial ecosystems to allow mitigation efforts to be established.

## Supplementary Material

Supplementary_Material_final_ycag140

## Data Availability

The datasets supporting the conclusions of this article are included within the article and its additional files or are available through the corresponding author upon reasonable request. The original sequencing data are available in the NCBI sequencing read archive under project accession number PRJNA1321686.
