# Supplementary material for: Selective shifts in mobile antibiotic resistance genes under carbamazepine exposure in wastewater microbiomes
Source: ISME Commun. 2026 May 22;6(1):ycag140. doi: 10.1093/ismeco/ycag140 (PMC13308717; doi:10.1093/ismeco/ycag140)
Supplement: Supplementary_Material_final_ycag140 [file supplementary_material_final_ycag140.pdf]

Supplementary Material for:

Selective shifts in mobile antibiotic resistance genes under carbamazepine exposure in wastewater microbiomes

Short title: Carbamazepine selects for mobile ARGs

Eda Deniz Erdem[1], Bing Li[2], Thomas U. Berendonk[1], Uli Klümper[1,#]

[1] Technische Universität Dresden, Institute of Hydrobiology, Dresden, Zellescher Weg 40, Germany

[2] Tsinghua University, Tsinghua Shenzhen International Graduate School, Institute of Environment and Ecology, Shenzhen, China

[#] corresponding author

Corresponding author

Dr. Uli Klümper (ORCID: 0000-0002-4169-6548)

Technische Universität Dresden,

Institute of Hydrobiology,

01062 Dresden,

Zellescher Weg 40,

Germany

E-mail: Uli.Kluemper@TU-Dresden.de

Phone: +49 351 463 43273

Supplementary methods:

Wastewater effluent community

The complex microbial community originated from the treated effluent of the Kreischa wastewater treatment plant (Dresden, Saxony, Germany) that was collected in June 2023. Two litres of the sampled effluent were filtered through 0.22 µm pore-size sterilised filters (Millipore). Three out of six filters used were placed in 50 mL tetrasodium pyrophosphate (TSPP) buffer and vortexed to facilitate the detachment of microorganisms. Harvesting the wastewater effluent microbial community via filtration and resuspending it in fresh medium was performed to reduce carry-over of dissolved compounds such as CBZ or antibiotics from the original effluent, thus allowing control of CBZ levels in the evolution experiment. The remaining filters were set aside for DNA extraction. The TSPP buffer was then added to 150 mL of LB broth in its entirety, along with the filters, to avoid a major loss of microbiome. This mixture was incubated for 24 hours at 30°C to obtain a liquid overnight culture of the microbial community to be used for evolution experiments and DNA extraction. The resulting DNA samples were denoted as Day 0 samples.

DNA extraction

All DNA extractions were carried out using the DNeasy PowerSoil Pro Kit (Qiagen, Hilden, Germany) per the manufacturer's protocol. In the cases where DNA was extracted from bacterial cultures, 2 mL of liquid culture at carrying capacity was collected, followed by centrifugation, a wash step, and the final resuspension in PBS. The isolated DNA was evaluated for its quantity and quality using a NanoDrop™ (ThermoScientific, Waltham, MA, USA) and stored at -20°C for further analyses.

16S rRNA gene-based qPCR

To assess if overall growth patterns of the community were affected by CBZ presence during the three-day experimental period, 16S rRNA gene copy numbers (gene copies/ng of DNA) in the samples were determined with SYBR Green-based quantitative PCR and used as an indicator of total bacterial abundance. A final concentration of 300 nM of the primer pair 338F (CCTACGGGAGGCAGCAG) - 518R (ATTACCGCGGCTGCTGG) [37] was used along with 10 µL of Luna® Universal qPCR Master Mix (New England Biolabs, Massachusetts, USA) and 5 µL of template DNA extract at 2 ng/µL in a final volume of 20 µL per reaction. A recombinant plasmid [38] was extracted and used as the qPCR standard at target concentrations of 10^8 - 10^2 copies per reaction. For each DNA sample, three technical replicates were set up in Real-Time qPCR in a C1000 Touch™ Thermal Cycler (BioRad, Hercules, CA, USA), and the cycling conditions were 44 cycles of 15 s at 95°C and 30 s at 60°C, preceded by an initial denaturation cycle of 95°C for 10 min. DNA isolates from treated wastewater effluent and initial augmented culture were analysed as well to assess the effect the pre-experiment protocol had on the bacterial load. Only the results without multiple peaks in the melting curve analysis and where the standard curve amplification efficiency was 0.9–1.1 and $R^2 \geq 0.99$ were accepted. Ten-fold dilution of template DNA revealed no PCR inhibition.

Microbial Community profiling

The composition and taxonomic diversity of pre-evolved and evolved microbial communities were evaluated by Illumina MiSeq amplicon sequencing of the 16S rRNA

gene. DNA samples at 10 ng/µL were transferred to the IKMB sequencing facility (Kiel University, Germany), where the bacterial 16S rRNA gene was sequenced using primers targeting the V3-V4 region (V3F: 5′-CCTACGGGAGGCAGCAG-3′ V4R: 5′-GGACTACHVGGGTWTCTAAT-3). Molecular analysis of the resulting sequences was carried out using the Mothur software package v.1.48.0[39] per MiSeq SOP[40]. Subsequently, OTUs were generated at 97% sequence similarity and taxonomically classified based on the RDP classifier. Sequencing depth of the samples varied between 403,338 reads at the highest and 4,896 at the lowest; this minimum value was then used for subsampling. Differences in the microbial composition of the samples were first analysed on the phylum level. Thereafter, OTU-based beta diversity of the sequences was assessed via non-metric multidimensional scaling (NMDS) by the Bray-Curtis dissimilarity index. The analysis of molecular variance (AMOVA) function of Mothur was used to determine the statistical significance of the spatial clustering of the samples, as indicated.

High-throughput quantitative PCR

The HT-qPCR protocol included the preparation of 100 nL of PCR reaction mixture with SmartChip TB Green Gene Expression Master Mix (Takara Bio, Japan), nuclease-free PCR-grade water, 300 nM of each primer, and 2 ng/µL DNA template. Cycling conditions were an initial denaturation step at 95°C for 10 min, after which 40 cycles of 95°C for 30 s and 60°C for 30 s followed. For each primer set, a melting curve analysis was performed. For the analysis of the resulting data, cycle thresholds (CT) above 31 were considered below the detection limit, and replicates where efficiencies were not between 1.8 and 2.2 were filtered out. The relative copy number of genes of interest normalized to the 16S rRNA gene was calculated using the ΔCT method[41],

where CT refers to the mean of three technical replicates. Non-detects were treated as zero values for relative abundance calculations. To avoid overinterpretation of sporadic detections, only genes detected in more than 50% of samples were retained for downstream analysis, while targets falling below the detection limit in most samples were excluded. This was in our dataset exclusively the case for $\textit{bla}_{\text{OXA-58}}$.

Supplementary tables

Table S1. qPCR primers used in this study based on Stedtfeld et al.[48]

Gene/Marker	Target	Forward Primer	Reverse Primer
16S rRNA gene	16S rRNA gene	GGGTTGCGCTCGTTGC	ATGGYTGTCGTCAGCTCGTG
aac(6')-Ib	Aminoglycoside	GTTTGAGAGGCAAGGTACCGTAA	GAATGCCTGGCGTGTTTGA
aac3-VI	Aminoglycoside	CGTCACTTATTCGATGCCCTTAC	GTCGGGCGCGGCATA
*aph*3-ib	Aminoglycoside	AACAGGTTTGGGAGGCGATG	CGCAACAAGCCTCTCCTGAA
*aph*6	Aminoglycoside	CCCATCCCATGTGTAAGGAAA	GCCACCGCTTCTGCTGTAC
bla_{CMY}	Beta Lactam	AAAGCCTCATGGGTGCATAAA	ATAGCTTTTGTTTGCCAGCATCA
bla_{CTX-M}	Beta Lactam	CGTACCGAGCCGACGTTAA	CAACCCAGGAAGCAGGCA
bla_{KPC2}	Beta Lactam	GCCGCCGTGCAATACAGT	GCCGCCCAACTCCTTCA
bla_{KPC3}	Beta Lactam	CAGCTCATTCAAGGGCTTTC	GGCGGCGTTATCACTGTATT
bla_{NDM}	Beta Lactam	GGCCACACCAGTGACAATATCA	CAGGCAGCCACCAAAAGC
bla_{OXA-48}	Beta Lactam	TGTTTTTGGTGGCATCGAT	GTAAMRATGCTTGGTTCGC
bla_{OXA-58}	Beta Lactam	GCAATTGCCTTTTAAACCTGA	CTGCCTTTTCAACAAAACCC
bla_{TEM}	Beta Lactam	CGCCGCATACACTATTCTCAG	GCTTCATTCAGCTCCGGTTC
bla_{VIM}	Beta Lactam	GCACTTCTCGCGGAGATTG	CGACGGTGATGCGTACGTT

*oqx*A	MDR	GAGTCAACCTACCTC CACTATCA	GCTGCGAGTTATCCA GCAG
*erm*B	MLSB	TAAAGGGCATTTAAC GACGAAACT	TTTATACCTCTGTTTG TTAGGGAATTGAA
*erm*F	MLSB	CAGCTTTGGTTGAAC ATTTACGAA	AAATTCCTAAAATCAC AACCGACAA
*mph*A	MLSB	CTGACGCGCTCCGT GTT	GGTGGTGCATGGCG ATCT
*qep*A	Quinolone	GGGCATCGCGCTGTT C	GCGCATCGGTGAAG CC
*qnr*S	Quinolone	CCACTTTGATGTCGC AGATCTTC	CCCTCTCCATATTGG CATAGGAAA
*sul*1	Sulfonamide	GCCGATGAGATCAGA CGTATTG	CGCATAGCGCTGGG TTTC
*tet*A	Tetracycline	GCTGTTTGTTCTGCC GGAAA	GGTTAAGTTCCTTGA ACGCAAAC
*tet*W	Tetracycline	ATGAACATTCCCACC GTTATCTTT	ATATCGGCGGAGAG CTTATCC
*dfr*A1	Trimethoprim	GGAATGGCCCTGATA TTCCA	AGTCTTGCGTCCAAC CAACAG
*dfr*A8	Trimethoprim	GGTCGCACCTGCATC GTTA	AGCGCCACCAATGAC GTAG
*van*A	Vancomycin	GGGCTGTGAGGTCG GTTG	TTCAGTACAATGCGG CCGTTA
*mcr*1	Other	CACATCGACGGCGTA TTCTG	CAACGAGCATACCGA CATCG
intI1	MGE	CGAACGAGTGGCGG AGGGTG	TACCCGAGAGCTTGG CACCCA
IncP_oriT	MGE	CAGCCTCGCAGAGC AGGAT	CAGCCGGGCAGGAT AGGTGAAGT
IncQ_oriT	MGE	TTCGCGCTCGTTGTT CTTCGAGC	GCCGTTAGGCCAGTT TCTCG
IncW_trwAB	MGE	AGCGTATGAAGCCCG TGAAGGG	AAAGATAAGCGGCAG GACAATAACG

IS*26*	MGE	ATGGATGAAACCTAC GTGAAGGTC	CGGTACTTAATCTGT CGGTGTTCA

Table S2. Strength of Spearman's rank correlation as described by Kuckartz et al.[53].

**Magnitude of R_s**	**Strength of correlation**
**$R_s > 0.70$**	Very strong correlation
**$0.50 < R_s < 0.70$**	Strong correlation
**$0.30 < R_s < 0.50$**	Moderate correlation
